# Effect of Embroidery Style on the Bandwidth of Textronic RFID UHF Transponder Antenna

**DOI:** 10.3390/s25020371

**Published:** 2025-01-10

**Authors:** Magdalena Nizioł, Piotr Jankowski-Mihułowicz, Mariusz Węglarski

**Affiliations:** 1 Department of Metrology and Diagnostic Systems, Rzeszów University of Technology, Wincentego Pola 2, 35-959 Rzeszow, Poland; 2 Department of Electronic and Telecommunications Systems, Rzeszów University of Technology, Wincentego Pola 2, 35-959 Rzeszow, Poland; wmar@prz.edu.pl

**Keywords:** RFID transponder, textronics, textile antenna, embroidery, embroidered antenna

## Abstract

The production of consumer electronics using electrically conductive materials is a dynamically developing sector of the economy. E-textiles (electronic textiles) are also used in radio frequency identification technology, mainly in the production of tag antennas. For economic reasons, it is important that the finished product is universal, although frequencies in radio systems have different values in different regions of the world. Therefore, the antenna bandwidth must be sufficiently wide so that the read range of the tag is maximally large for all frequencies of the specified band. The bandwidth of an antenna depends on its type and geometric dimensions, but this parameter can also be influenced by the way a given type of antenna is made. The authors prepared samples of embroidered RFID tag antennas for the UHF band using various types of embroidery. Then, its impedance and the read range of the tag were examined in order to determine the exact influence of the type of embroidery on the parameter of interest (antenna bandwidth). The results obtained during the research indicate the influence of different embroidery styles is present; however, that influence is not significant.

## 1. Introduction

### 1.1. Purpose of the Work

Technological development is largely linked to changing consumer demands. Over the years, there has been an increasing emphasis on miniaturizing devices while maintaining a certain durability and, where possible, ensuring wireless operation. In order to meet such requirements, new fields of science have begun to develop, and one of them is textronics, which is a combination of, among others, electronics and textiles [1].

Nowadays, consumer electronics are increasingly integrated with electrically conductive textiles, which significantly expands the range of applications of individual devices [2,3,4,5]. One of the sectors of the economy in which e-textiles (combination of embedded electronics and textiles) are becoming increasingly important is the technology of radio frequency identification (RFID). Conductive materials (e.g., threads, fabrics) are used in the production of transponder antennas [6,7,8,9,10,11], thanks to which it is possible to integrate finished products with, e.g., items of clothing in a relatively easy and aesthetic manner. RFID tags with such antennas are widely used in other industries, such as health care [12,13,14] or the military [15,16].

A significant part of transponders is designed to operate in the UHF (ultra-high frequency) band [10,17,18,19]. Transponders for this band prevail over their counterparts in the low-frequency (LF) and high-frequency (HF) bands mainly in terms of the read range from about 10 m in the case of passive tags, to even 100 m in the case of active tags, with the appropriate selection of the antenna being of great importance.

The simplest type of antenna for UHF band RFID tags is a classic dipole antenna. By definition, however, it is a narrowband antenna, and in the UHF band, different frequencies are allocated to RFID devices depending on the region of the world, e.g., for the region covering Europe (region 1, according to frequency allocation by International Telecommunication Union), it is 862–870 MHz and for Asia (region 3), it is 950–956 MHz [20]. Due to this frequency dispersion, the use of narrowband antennas is uneconomical, as a tag designed to operate in one of the previously listed regions may be incompatible with RFID systems in another region, which may necessitate the production of additional solutions.

From an economic point of view, the ideal situation is to develop a solution with a wideband antenna so that the tag works effectively regardless of location. An example of such an antenna can be the bow-tie antenna, which is still an object of interest for engineers and scientists [21,22,23,24,25,26,27,28].

A bow-tie antenna is a planar antenna. Making it in the form of a textile antenna requires embroidering a relatively large surface of the radiator, which translates into a significant amount of material used (e.g., conductive thread). In mass production, this can generate considerable costs, which can be reduced by optimizing the antenna embroidery process.

The main parameter on which the authors focus their attention is the bandwidth of the antenna, so that after integrating it with the chip, the finished tag can effectively work in the frequency band from 860 to 960 MHz (UHF band coverage for all regions of the world). The authors are looking for a relationship between this parameter and the style of embroidery used to produce the model antennas. It was hypothesized that a change in the style and density of embroidery would cause a change in bandwidth, but a properly optimized embroidery process could reduce the amount of material used without significantly deteriorating the parameters of the tested antenna.

Similar studies have been found in the literature, but they either do not directly concern antennas [29], or focus on the relationship between the type of embroidery and other parameters, e.g., the shape of the antenna [30]; tag read range [31,32,33,34,35]; impedance [34]; gain, efficiency and radiation pattern [36,37,38,39]; reflection coefficient [36,37,38,40] (indirectly related, but not exhaustive of the subject under study).

### 1.2. Textile UHF RFID Transponder Antenna

**Figure 1:**
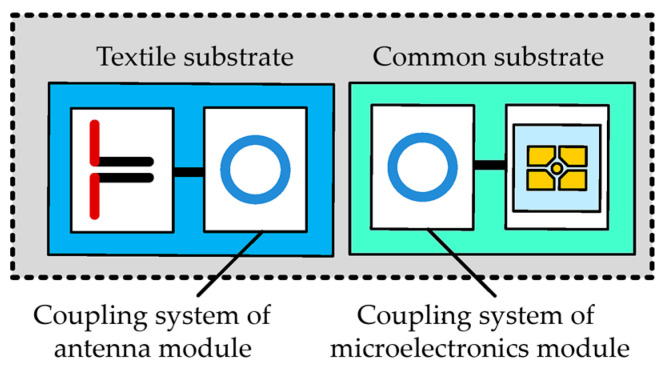
Block diagram of textronic RFID transponder (RFIDtex tag).

The research team of the co-authors of the article has been dealing with the subject of RFID for many years, and the result of their research is, among others, a patent (patent no PL 231291 B1 Polish Patent Office) concerning a textronic tag (Figure 1) [41]. According to the idea behind this solution, the antenna and chip are not galvanically connected. Instead of a physical connection carried out, e.g., by means of a conductive thread from which the tag antenna is sewn, two coupling circuits in the form of loops are used.

A solution of a textronic tag for the UHF band with a dipole antenna was developed and tested in many aspects [42,43,44]. As part of this article, the dipole antenna is replaced by a wideband bow-tie antenna. In the design, the length of the antenna corresponds to the length of the classic dipole being replaced. An increasing bandwidth is ensured by the expansion of the radiator. The antenna is connected to the chip in a manner analogous to the previous solution, i.e., by means of a loop with a diameter of 5.5 mm ensuring inductive coupling with the microelectronic circuit.

**Figure 2:**
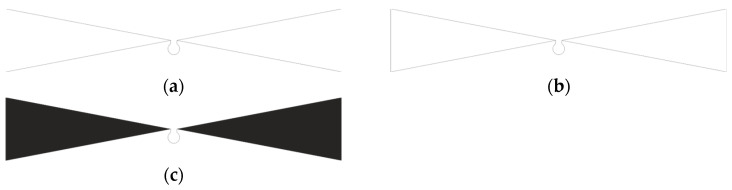
Geometry of designed antennas: (**a**) open contour; (**b**) closed contour; (**c**) planar.

Due to the fragment of the hypothesis presented in Section 1.1 about the limitation of the use of material to create an antenna, in the experiments, in addition to the planar model, contour models were also taken into account. The geometry of the developed models is shown in Figure 2.

Contour models were prepared for the analysis, including open (a) and closed (b), as well as a planar model (c), under which, by means of the style and density of embroidery, the degree of filling of the radiator will realistically be changed.

To ensure the proper operation of the RFID tag, a proper impedance match between the antenna and the chip is necessary. Both the antenna impedance (Equation (1)) and the impedance of the chip (Equation (2)) are expressed in complex form and are conjugates of each other. The quality of this conjugation is expressed numerically by power transfer coefficient *τ* (Equation (3)).

*Z_TA_* = *R_TA_* + *jX_TA_*, (1)

*Z_TC_* = *R_TC_* + *jX_TC_*, (2)

(3) $$\tau =\frac{4Re\left(Z_{TA}\right)Re(Z_{TC})}{{\left(Re\left(Z_{TA}+Z_{TC}\right)\right)}^{2}+{\left(Im\left(Z_{TA}+Z_{TC}\right)\right)}^{2}},$$

In Equations (1) and (2), the symbols *R_TA_* and *R_TC_* stand for antenna resistance and chip resistance, respectively, while *X_TA_* and *X_TC_* represent reactance. Symbols *Z_TA_* and *Z_TC_* stand for antenna and chip impedance.

## 2. Materials and Methods

**Figure 3:**
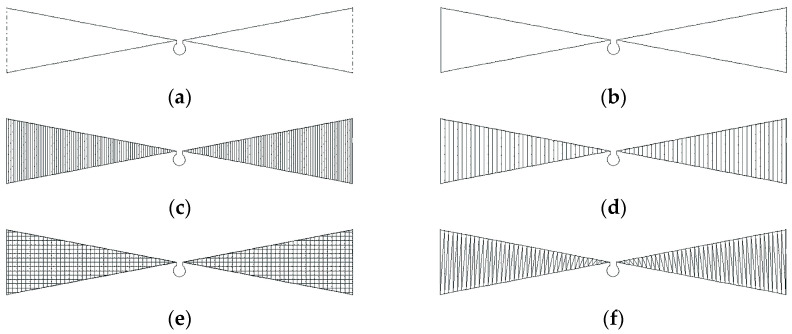
Models prepared in PE DESIGN 11: (**a**) model MN1—open contour; (**b**) model MN2—closed contour; (**c**) model MN3—planar ver. 1; (**d**) model MN4—planar ver. 2; (**e**) model MN5—planar ver. 3; (**f**) model MN6—planar ver. 6.

In order to create samples of the analyzed antenna, its geometry was first implemented into the PE DESIGN 11 program, which is dedicated to Brother (Brother Industries, Ltd., Nagoya, Japan) embroidery machines. In this software, in addition to designing the pattern and configuring key parameters of the embroidery, there is also an option to simulate the embroidery process. The view of the first models prepared for embroidery is shown in Figure 3.

The first two samples show just the outline of the antenna. The next 4 are a filled model, but the samples differ in the style of stitch filling the surface of the radiators and the density of the stitch. The exact sewing parameters of individual models are presented in Table 1.

The MN3 pattern most closely represents the full filling of the radiator surface. Each successive pattern is realized with a lower density, resulting in less use of the conductive thread. However, in the case of patterns with vertical seams, there is a risk of too weak an electrical connection between successive seams, so the MN5 pattern was implemented in the form of a grid; horizontal stitching should significantly improve the electrical connections between successive sections of thread.

**Figure 4:**
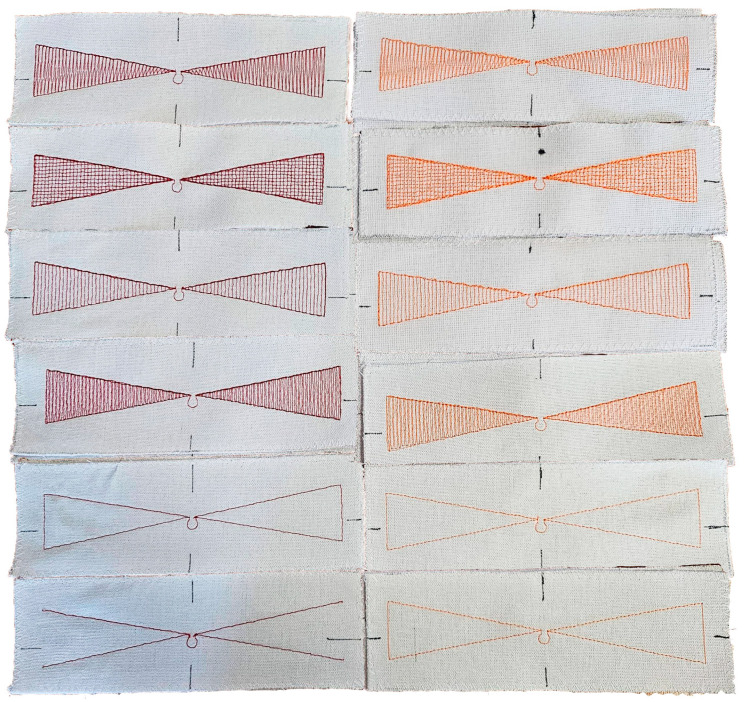
Photograph of sewn samples (red ones—thread B; orange—thread A).

The designs shown in Figure 3 were embroidered using the BROTHER INNOV-IS V3 (Brother Industries, Ltd., Nagoya, Japan) embroidery machine. The conductive thread was placed in the bobbin (bottom thread), while the upper thread was a non-conductive polyester sewing thread. All antennas were embroidered at the speed of bobbin 350 rpm, with the tension of the thread set at level 9. Conductive threads were used to create the samples, the basic parameters of which are presented in Table 2. All patterns are embroidered on linen fabric. The appearance of the finished samples is shown in Figure 4.

**Figure 5:**
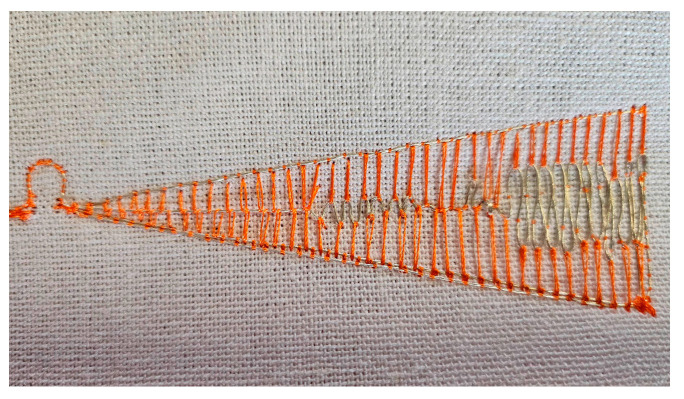
Photograph of a deformed sample (model MN6).

The threads (the ones marked as A and B) used, in addition to parameters presented in Table 2, also differ in stiffness, which at the production stage can affect the final effect of the embroidery process—the stiffer the thread, the more difficult it is to arrange it into more complex patterns. In addition, the number of needle insertions should be properly selected—too small a number may cause deformation of the pattern, which was the case with the MN6 pattern (Figure 5).

Although seemingly the pattern can be considered completely unsuccessful and the sample useless, the antenna was eventually used for research, because part of the radiator is embroidered (patch at the edge) and it has an electrical connection to the contour and coupling circuit.

**Figure 6:**
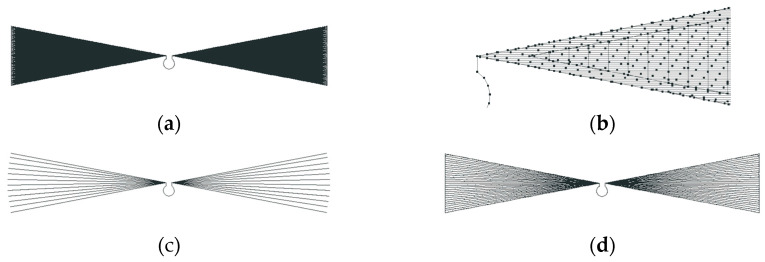
Models prepared in PE DESIGN 11: (**a**) model MN8—planar ver. 5; (**b**) model MN8—closeup; (**c**) model MN9—planar ver. 6; (**d**) model MN10—planar ver. 7.

To make the research more comprehensive, a decision was made to extend the experiments with additional formulas (Figure 6) and threads (marked as C and D in Table 2). Additional samples were sewn with threads with C and D markings on the same substrate material.

**Figure 7:**
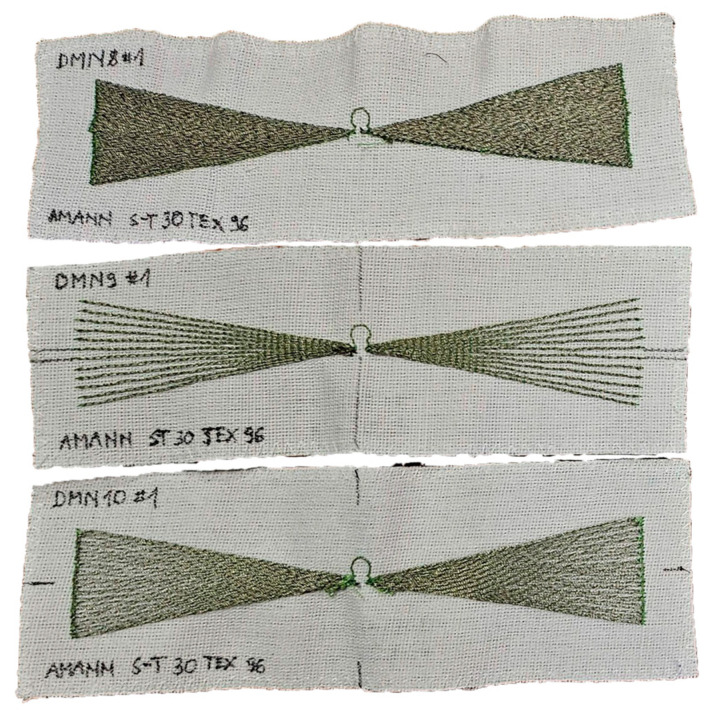
Photograph of sewn samples (selected models from group D).

Finally, a total of 42 samples divided into 4 groups (depending on the conductive thread used) were prepared for testing. However, the main object of interest was the groups A and B containing MN1–MN6 models. Groups C and D (the appearance of selected samples is shown in Figure 7) complementing the analysis contain MN1, MN4 and MN5 models from the basic pool of projects and additionally, MN8, MN9 and MN10 models from the additional pool (group D only). At this point, it should also be mentioned that group C was used only for some of the experiments.

**Figure 8:**
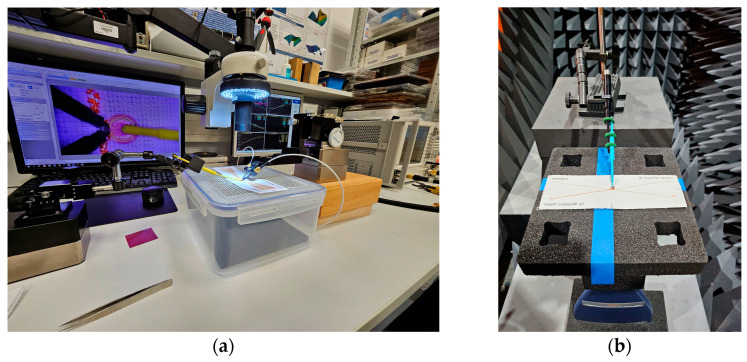
Photographs of the measurement stands: (**a**) impedance measurements; (**b**) anechoic chamber.

The prepared samples were used for two stages of research—first, the impedance of the model antennas was measured, and then the read range of the tag with the model antenna was examined. The appearance of the measuring stations used during the tests is shown in Figure 8.

**Figure 9:**
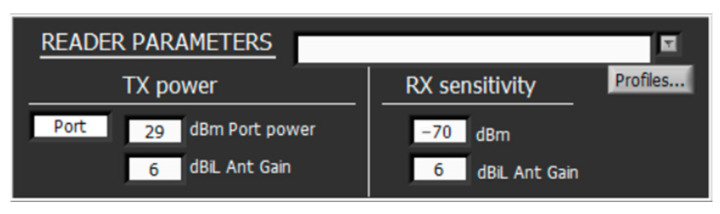
Reader parameters in Tagformance.

For impedance measurements (Figure 8a), a Keysight (Keysight Technologies, Inc., Santa Rosa, CA, USA) PNA-X N5242A vector network analyzer with attached PacketMicro DPSS201505 SS05–0053 probes was used. A microscope was additionally used to check the correct attachment of the probe to the measuring circuit. The read range measurements were carried out in the Microwave Vision Group (Paris, France) anechoic chamber equipped with the Voyantic (Voyantic Ltd., Helsinki, Finland) Tagformance Pro measurement system. This system is a comprehensive combination of hardware and software (Tagformance Pro UHF, currently in version 14.0) dedicated to RFID tag measurement. Measurements were made in accordance with the ISO IEC 18000-63 standard. The set parameters of the reader are shown in Figure 9.

## 3. Results

The aim of the study was to determine whether the style of embroidery used to produce a planar antenna affects its parameters, in particular the bandwidth. Changes in the bandwidth value can directly affect the performance of the tag under certain conditions.

**Figure 10:**
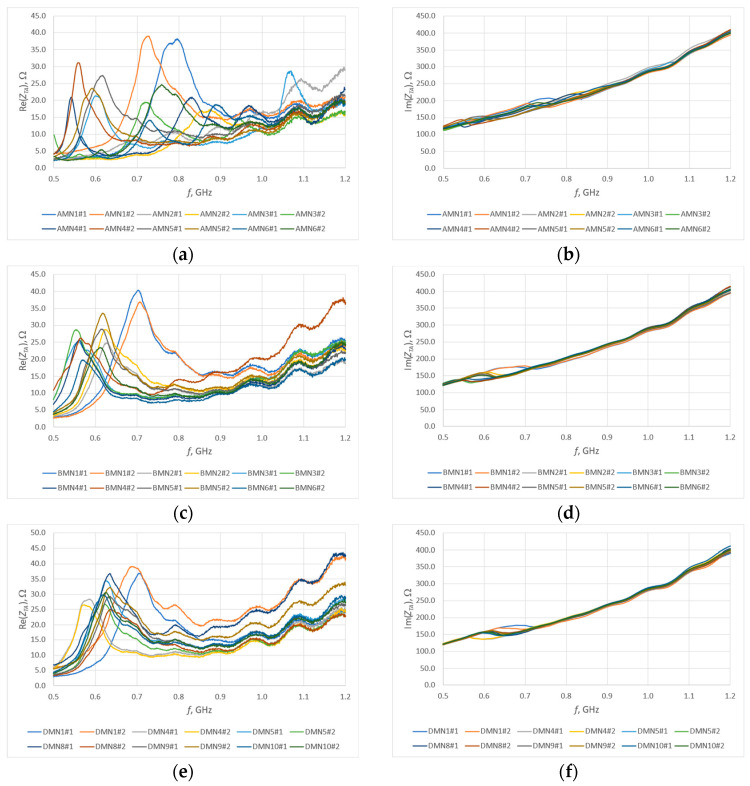
Impedance of the model antennas: (**a**) group A, real part; (**b**) group A, imaginary part; (**c**) group B, real part; (**d**) group B, imaginary part; (**e**) group D, real part; (**f**) group D, imaginary part.

In order to determine the bandwidth, the impedance of the model antenna must first be measured. The impedance measurements of individual samples were made in the frequency range of 0.5–1.2 GHz. The results obtained are presented in Figure 10.

Between the individual models, the change in the antenna resonance frequency is visible (dispersion between about 550 and 700 MHz). The values for planar models are similar, but for contour models, and in particular for open contour models, the value is much higher. This is mainly related to changes in the *R*, *L* and *C* parameters of the antenna. The impedance value also changes, with the trend of changes being analogous. An important observation during the analysis is the significant similarity in the shape of the waveform and the measured values in the case of planar models, which may suggest that there is no need to fully embroider the radiator to achieve similar antenna parameters.

The observed differences between samples of the same model result directly from the production process of the model antenna. During sewing, not only the thread from which the antenna is sewn, but also the substrate material is subjected to tension. Each deformation causes some dispersion of antenna parameter values—the largest differences are visible in the case of group A samples; in other cases, the samples are very similar to each other and the differences are negligibly small.

**Figure 11:**
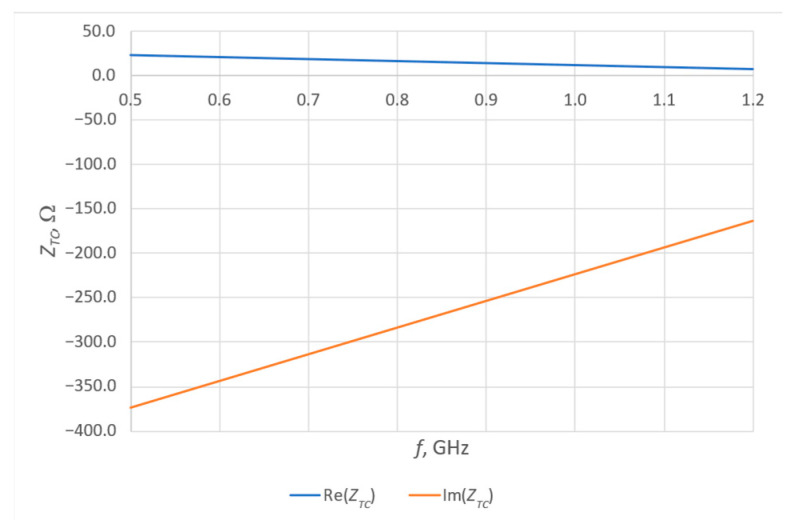
SL3S1214 UCODE 7m chip impedance.

**Figure 12:**
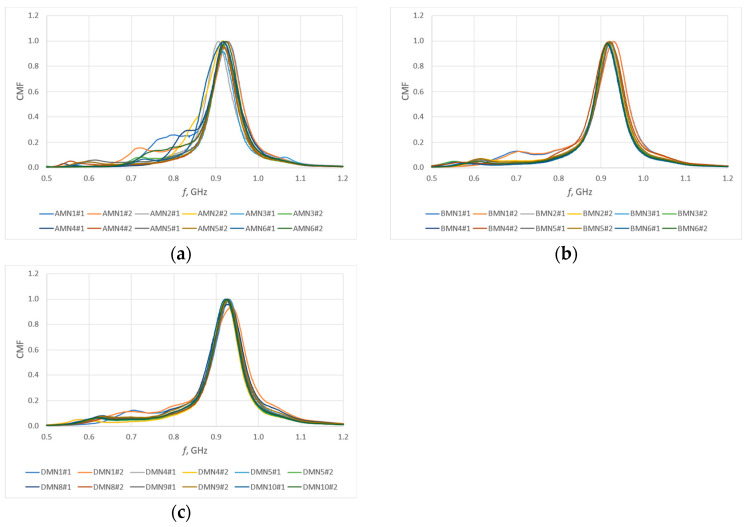
Calculated CMF: (**a**) calculations for group A; (**b**) calculations for group B; (**c**) calculations for group D.

A good parameter from which to determine the bandwidth of an antenna can be the power transfer coefficient, often also referred to as conjugate match factor (CMF) (Equation (3)). This coefficient describes the quality of the antenna’s fit to the chip, and its value ranges from 0 (no match) to 1 (perfect match). The impedance of the chip (Figure 11)—SL3S1214 UCODE 7m in case SOT886 manufactured by NXP Semiconductors, Eindhoven, The Netherlands—necessary to determine the CMF was previously determined by numerical calculations (linear regression on data from chip datasheet). The selected chip operates in passive (no additional power supply) mode and exactly this solution is tested. Figure 12 shows the CMF values calculated for the model antennas as a function of frequency.

It should be noted that the impedance of the chip changes with frequency. In addition, the imaginary part takes on relatively large values, which consequently slightly increases the challenge of perfect antenna matching.

**Figure 13:**
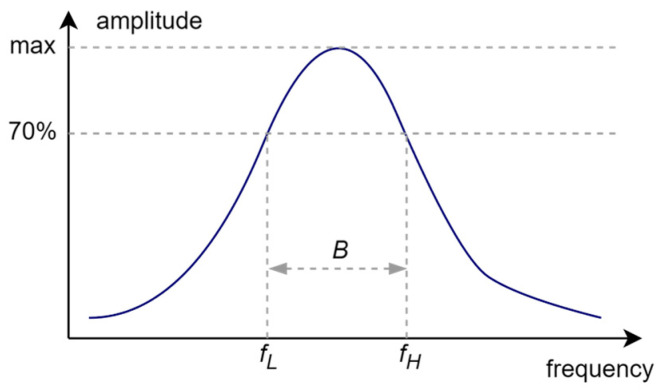
Methodology of determining model antenna bandwidth.

**Figure 14:**
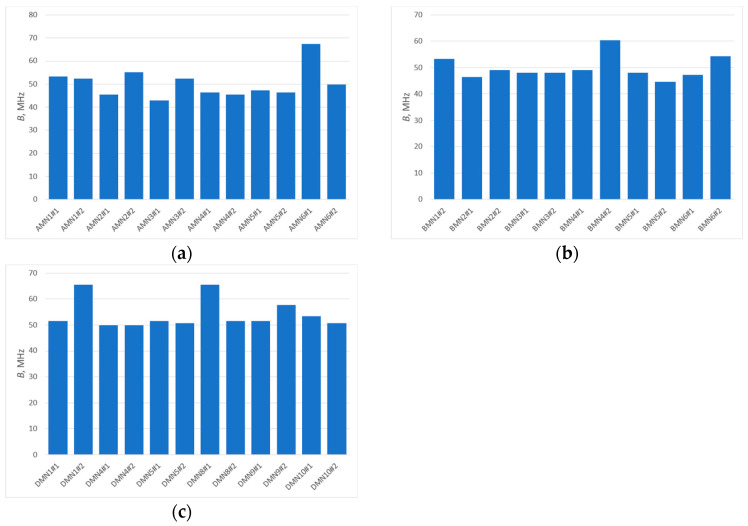
Graphical representation of model antenna bandwidth (determined from CMF) values for: (**a**) samples from group A; (**b**) samples from group B; (**c**) samples from group D.

The analysis of the presented courses shows a large convergence of the obtained results. An important observation, nevertheless, is the increase in resonant frequency relative to the values observed in the antenna impedance plots. This is due to the fact that the presented results are for a resonant circuit consisting of an antenna and a chip. However, from the point of view of the purpose of this publication, it is important whether the bandwidth is changed, not the CMF that was used to determine it. This bandwidth *B* (Figure 13) is determined as the difference between the upper (*f_H_*) and lower (*f_L_*) frequency at which the parameter (in this case CMF) reaches 70% of its maximum value (within the assumed range of best fit, the antenna will provide the most effective operation of the tag). The results obtained are presented in a graphic form in Figure 14.

The results obtained are characterized by a certain spread of values. By determining the standard deviation (Equation (4)) and then the relative standard deviation (Equation (5)) from the obtained results (Table 3), a spread around the mean in the range from 9% (group B) to 13% (group A) is obtained. It is worth noting that in the results obtained, however, there are single values that differ significantly from the others. Comparison of the questioned values with the values for the second samples of a given model shows a relatively large discrepancy; therefore, these values can be treated as parasitic errors caused by, for example, deterioration of the coupling between the antenna and the chip coupling system during the measurement (shifted chip substrate). Eliminating these values from the analysis will cause the spread of bandwidth values of the model antennas to be even smaller, and this leads to the conclusion that the type of embroidery has a limited impact on the antenna’s bandwidth.

(4) $$s=\sqrt{\frac{\sum {\left(B_{i}-B_{av}\right)}^{2}}{n-1}},$$

(5) $$s_{rel}=\frac{s\cdot 100}{B_{av}},$$

where *B_i_* stands for individual sample bandwidth, *B_av_* is the average bandwidth for all samples within group and *n* is the number of samples in the group.

**Figure 15:**
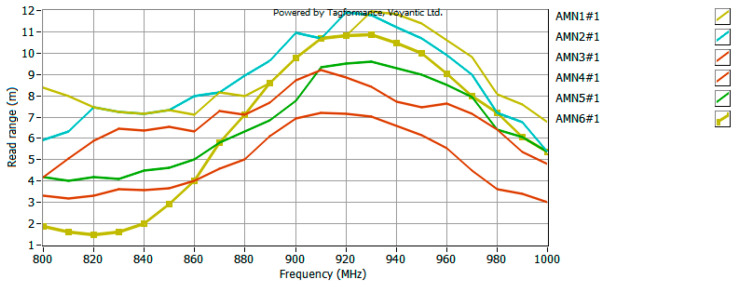
Read range of UHF RFID tag with model antenna—data obtained for group A.

**Figure 16:**
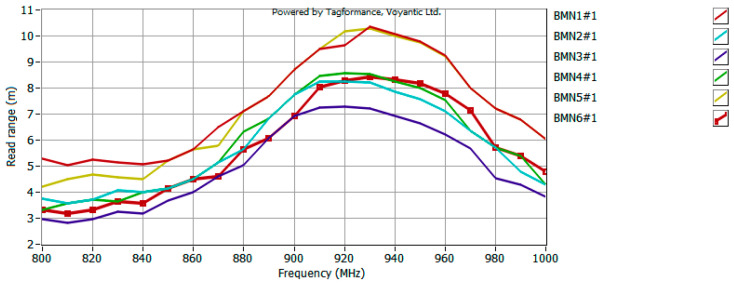
Read range of UHF RFID tag with model antenna—data obtained for group B.

**Figure 17:**
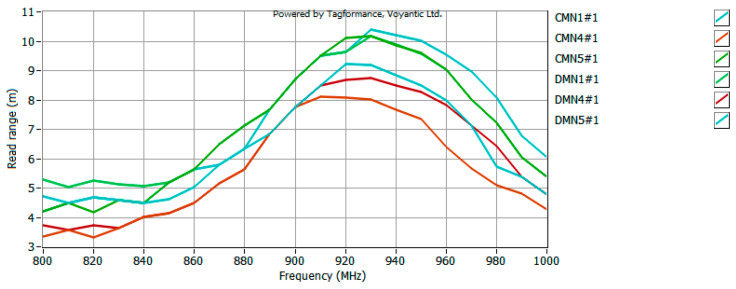
Read range of UHF RFID tag with model antenna—data obtained for groups C and D.

The model antennas were prepared for the UHF RFID tag, so the last stage of the research was to measure the read range of such a tag in the anechoic chamber. The waveforms recorded in the UHF Tagformance software (for selected samples) are shown in Figure 15, Figure 16 and Figure 17.

**Figure 18:**
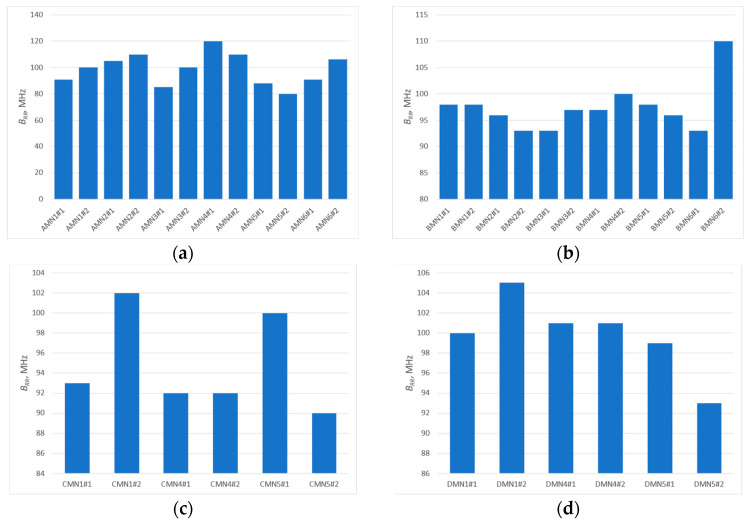
Graphical representation of model antenna bandwidth (determined from read range) values for (**a**) samples from group A; (**b**) samples from group B; (**c**) samples from group C; (**d**) samples from group D.

Analyzing the obtained plots, it can be seen that the maximum read range is shifted towards the upper limit of the UHF band. This could be expected from an analysis of the CMF graphs, since the frequency for which the antenna is best matched (CMF equal to or close to 1) is also shifted toward the upper limit of the band. However, the bandwidth guaranteed by the bow-tie antenna ensures the operation of the tested tag in the entire analyzed band (860–960 MHz). The analysis of the obtained plots in terms of bandwidth (*B_RR_*) values of the tested antennas (carried out analogously to the analysis on the basis of CMF) (Figure 18) confirms the previous conclusions. The difference between the patterns is not very significant, i.e., a relative standard deviation (calculation results shown in Table 4) between 4% for part of group D (only three models from the basic pattern pool were examined) and 12% for group A. Therefore, there is no need to fully embroider the designed geometry. The type of embroidery can be selected so as to minimize the amount of material used, and thus reduce the cost of the antenna production.

While the results obtained are satisfactory, it is possible to significantly improve them (especially in the context of the bandwidth) thanks to better impedance matching of the model antennas. Then, it would be possible to bring the curve to a complete flattening in the entire band.

## 4. Discussion

The aim of the study was to investigate the effect of the style of embroidery used to create textile planar antennas on the bandwidth. The motivation for undertaking the research was to determine the possibility of optimizing the economic aspect of the antenna production process. The cost of conductive threads, especially for mass production, is significant, so the amount of material used is extremely important.

In order to meet the assumed goal, several versions of the bow-tie antenna were first prepared, including an antenna model in the form of an open contour (MN1), a closed contour (MN2) and planar models with different method and density of radiator filling (MN3–MN10). In addition, different conductive threads were used to create the samples, so that the tests carried out would be more comprehensive.

The first stage of the research consisted of measurements of the impedance of model antennas carried out in the frequency range from 0.5 to 1.2 MHz. The obtained results were analyzed within individual groups of samples: A (thread Syscom Liberator 40), B (thread Plug & Wear PW018A) and D (thread AMANN Silver-tech). The largest changes in impedance were observed between the MN1 and MN2 models, and the planar models, which is obvious, if only because of the differences in the amount of conductive thread used and the indirectly resulting changes in the values of parasitic parameters in the antenna. In the case of planar models, the observed changes in impedance between different embroidery patterns were not characterized by such a significant spread.

On the basis of the measured impedances of the model antennas, the conjugate match factor (CMF) was determined, which was then used to determine the bandwidth of the model antennas. CMF plots have much more similarity to each other (within a given group of samples and between groups) than impedance plots. A change in the resonant frequency relative to the impedance plots is observed—this is due to the impedance of the chip. Analysis of the bandwidth results obtained at this stage showed differences in the values of this parameter for different patterns, but these differences are in the order of 10%. A change at this level in many cases can be considered negligibly small.

The last stage of the research was to measure the read range of the RFID tag on the UHF band with model antennas. All the tested antennas met the basic requirement, i.e., the tag works in the entire band from 860 to 960 MHz (even though the CMF at the lower end of the band deviates significantly from 1), although in the specified frequency range, the value of the read range changes (the trend of changes coincides with CMF plots, which means that as the antenna matching changes, the read range changes as well). The results obtained could be improved if the impedance matching of the model antennas was improved.

An important observation regarding the obtained results is the change in the value of the frequency for which antenna matching is best (shift towards the upper limit of the analyzed band). This is due, among other things, to the parasitic capacitances found in the model antennas. This observation is consistent with the conclusions of other researchers, whose work is referred to in Section 1.1.

In Section 1.1, a hypothesis is presented that a change in the style and density of embroidery will cause a change in bandwidth, but a properly optimized embroidery process can reduce the amount of material used without significant deterioration of the parameters of the tested antenna. On the basis of the analyses carried out, this hypothesis can be considered confirmed. The stitch patterns used in the study had a negligible effect on changes to the bandwidth. Much more important in the context of the results obtained is ensuring the best possible impedance matching of the antenna.

## 5. Conclusions

Textile antennas are an important part of the growing new fields of the economy and are of interest to many researchers. In this paper, the authors analyze the effect of embroidery style on the bandwidth of the bow-tie antenna of a textronic UHF RFID tag. Different versions of planar antennas have been investigated; the degree of embroidery of the radiator depends on the style of embroidery. Analyzing the data collected during the experiments, it was found that there are noticeable differences in the value of the parameter of interest (antenna bandwidth), but these differences do not have a significant impact on the performance of the tag with which the model antenna is integrated.

## Figures and Tables

**Table 1:** Detailed information about used styles of embroidery.

Model	Stitch Style	Density	Stitching Direction
MN1	Running Stitch	2 mm	-
MN2	Running Stitch	2 mm	-
MN3	Fill Stitch	1.0 line/mm	90 deg
MN4	Fill Stitch	0.5 line/mm	90 deg
MN5	Net Fill Stitch	2 mm	0 deg
MN6	Satin Stitch	1 line/mm	90 deg

**Table 2:** Conductive thread parameters.

Symbol	Manufacturer	Brand Name	Material	Thread Resistivity, Ω/m
A	Syscom Advanced Materials, Inc., Columbus, OH, USA	Liberator 40	Silver coated vectran	3.28
B	Plug&Wear, Florence, Italy	PW018A	Silver plated nylon	300
C	Sparkfun Electronics, Niwot, CO, USA	DEV-11791	Stainless steel	91.86
D	AMANN Group, Bönnigheim, Germany	Silver-tech 30 tex 96	Silver coated polyamide/polyester	85

**Table 3:** Standard deviation and relative standard deviation calculated for bandwidth determined from CMF.

Group of Samples	*s*, MHz	*s_rel_*
A	6.57	13.05%
B	4.46	8.95%
D	5.74	10.59%

**Table 4:** Standard deviation and relative standard deviation calculated for bandwidth determined from read range.

Group of Samples	*s*, MHz	*s_rel_*
A	11.97	12.11%
B	4.56	4.68%
C	4.92	5.18%
D	3.92	3.93%

## Data Availability

All calculated and measured data will be provided upon request to the correspondent authors by email with appropriate justification.
